# Green Technique for Producing Carbon-Based Catalysts for Cellulose Hydrolysis

**DOI:** 10.3390/ma18215031

**Published:** 2025-11-05

**Authors:** Siqi Deng, Kaixun Yao, Manabu Kodama, Oi Lun Li, Nozomi Takeuchi

**Affiliations:** 1 Department of Electrical Engineering, Tohoku University, Sendai 980-8579, Japan; 2 Department of Electrical and Electronic Engineering, Institute of Science Tokyo, Tokyo 152-8552, Japan; 3 Department of Mechanical Engineering, Institute of Science Tokyo, Tokyo 152-8550, Japan; 4 School of Materials Science and Engineering, Pusan National University, Busan 46241, Republic of Korea

**Keywords:** acid-free sulfonation, carbon nanotubes, solid acid catalyst, cellulose hydrolysis, sulfate radicals

## Abstract

Sulfonated carbon catalysts, as a type of solid protonic acid, have been widely recommended for various applications. However, syntheses of them typically require strict conditions, posing challenges in efficiency and environmental impact. Herein, we report a rapid, acid-free method to introduce sulfonic and sulfate ester groups onto carbon nanotubes (CNTs) by simply stirring them in an aqueous sodium persulfate solution (Na_2_S_2_O_8_) at room temperature. Within 45 min, the treated CNTs reached sulfur-containing acid densities up to 0.34 mmol g^−1^ without thermal treatment and hazardous reagents. The resulting catalyst demonstrated effective performance in terms of cellulose hydrolysis, attaining 31.6% conversion and 23.2% glucose yield. The process requires only the energy input of magnetic stirring, underscoring its environmental and practical advantages. This simple approach provides a sustainable and cost-effective alternative for the preparation of carbon-based catalysts, offering significant potential for biomass conversion and other green-chemistry applications.

## 1. Introduction

Sulfonated carbon catalysts, known as solid protonic acids, have been extensively studied for diverse catalytic reactions [1,2,3,4], Their advantages include high thermal stability [5], recyclability [6,7,8], and strong protonic acidity, which make them particularly suitable for cellulose depolymerization [3,9]. However, conventional sulfonation methods generally rely on concentrated sulfuric acid and high-temperature treatment, generating hazardous waste and consuming significant energy [3], such as impregnating precursor with concentrated H_2_SO_4_, followed by pyrolysis under dry nitrogen at 300 °C for 1 h [5]. These harsh conditions pose significant challenges for sustainable catalyst manufacturing and limit process scalability.

To address these limitations, recent studies have investigated greener sulfonation pathways, including the use of *p*-toluenesulfonic acid (TsOH) as both carbon precursor and sulfonating agent [10,11], sulfur-containing gas functionalization [12], and plasma-assisted activation of dilute sulfuric acid [13,14]. Despite their partial success, such methods still involve corrosive reagents, high energy input, or complex equipment [15]. A truly acid-free and low-energy route for preparing sulfonated catalysts remains highly desirable. Recent advances in green oxidation chemistry have highlighted the potential of persulfate salts as clean, water-soluble oxidants capable of producing sulfate radicals (SO_4_•^−^) under mild conditions [16,17]. Over the past few decades, extensive research has been conducted on Na_2_S_2_O_8_ activation methods, including base treatment, UV irradiation, heat treatment, and carbon-based activation [16,17,18,19]. In the carbon-based activation, while carbon-based activation has been extensively studied in pollutant degradation [17,18], its potential for surface functionalization of carbon materials for catalytic applications remains underexplored.

Several studies have reported sulfonation of carbonaceous materials under sulfuric acid conditions, typically achieving 15–40% cellulose conversion and 10–30% glucose yield [13,14]. Although these acid-based methods effectively introduce –SO_3_H groups to produce high-performance catalysts, they require concentrated acids and elevated temperatures or significant electrical energy input, resulting in substantial waste generation. Recent reports have suggested that SO_4_•^−^ can activate carbon sites and facilitate the grafting of sulfur-containing functional groups onto the carbon framework [20,21]. Herein, we present a simple, acid-free strategy for sulfonating CNTs by stirring them in aqueous Na_2_S_2_O_8_ at room temperature. The carbon surface mediates persulfate activation to generate oxidative radicals, enabling the formation of sulfonic (–SO_3_H) and sulfate ester (–OSO_3_H) groups without thermal treatment. The catalytic performance of the treated CNTs was evaluated, and their surface elemental composition was analyzed to confirm the effectiveness of this treatment.

## 2. Experimental Section

### 2.1. Materials and Sulfonation Procedures

**Figure 1:**
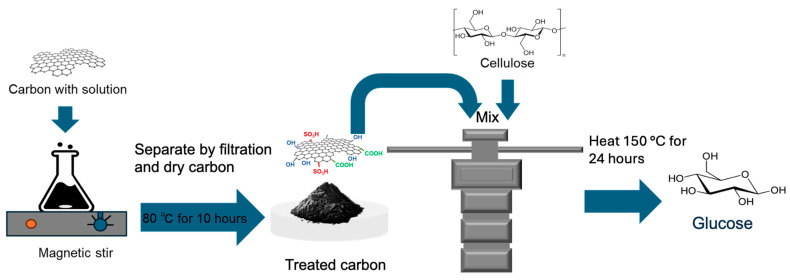
Schematic of the treatment process.

The experimental conditions are listed in Table 1, and the experimental procedure is illustrated in Figure 1. Commercial CNTs (Multiwall CNTs, JEIO 6A, Incheon, Republic of Korea; 3~5 walls, average diameter: 4~6 nm; length: 50~150 um; purity: >96.5 wt%) were stirred in 50 mL aqueous Na_2_S_2_O_8_ solutions with concentrations of 0.1, 0.25, or 0.5 M at room temperature (20 °C) for 10, 20, or 45 min. For comparison, a control sample was prepared by hydrothermal sulfonation in 18 M H_2_SO_4_ at 150 °C for 10 h, following a conventional acid-based procedure. After treatment, the samples were collected by filtration, thoroughly washed with distilled water, and dried overnight at 80 °C. The pH of each solution was monitored using a pH meter (Hanna HI1131, Chiba, Japan) which was calibrated using standard buffer solutions ranging from pH 1.00 to 12.00 before each measurement. Each experiment, including catalyst synthesis and cellulose hydrolysis, was performed in triplicate.

### 2.2. Characterization

X-ray photoelectron spectroscopy (XPS) measurements were performed on a PHI 5000 VersaProbe III (ULVAC-PHI, Chigasaki, Japan) system using a monochromatic Al Kα source (1486.6 eV) under an ultrahigh-vacuum of <1 × 10^−6^ Pa. The photoelectron take-off angle was 45°. Binding energies were calibrated against the C 1s peak at 284.8 eV to correct for charging effects. For the survey scans, the resolutions were set as 0.4 eV, 0.1 eV and 0.2 eV were set for element-specific scans. Survey and high-resolution spectra were fitted using Multipak (9.2.0.5) software. Elemental sulfur content was measured by CHNS elemental analysis using a combination of an HSU-20 instrument (Yanako, Kyoto, Japan) and ICS-1100 instrument (Thermo Fisher, Waltham, MA, USA) system with a combustion cube temperature of 950 °C, while oxygen content was determined using a Vario Micro Cube (Elementar, Langenselbold, Germany) with a combustion cube temperature of 1150 °C. The total acid density was quantified by the Boehm titration method with an automatic titrator (HI 901C1, Hanna Instruments, Chiba, Japan). The carbon sample will be mixed with 40 mL 0.01 M NaOH solution and use magnetic stirrer to stir the mixture for 10 h. The total oxygen-containing functional groups on the carbon surface were determined by neutralizing with 0.01 M HCl solution. Surface morphology was examined by transmission electron microscopy (TEM; JEOL JEM-2010F, Tokyo, Japan) and scanning electron microscopy with energy-dispersive X-ray spectroscopy (SEM-EDS; Hitachi TM4000 equipped with Bruker QUANTAX 75-60, Tokyo, Japan). Nitrogen adsorption–desorption isotherms were recorded on a BELSORP mini X system (Microtrac MRB, York, PA, USA) to determine the specific surface area and pore size distribution of the samples. Before the BET measurement, a 100 mg carbon sample was degassed under vacuum to 120 °C for 24 h. Fourier Transform Infrared Spectroscopy (FTIR) spectra of original CNTs and treated CNTs were collected using a JASCO FT/IR-6600 (Tokyo, Japan) spectrometer in the range of 1000–4000 cm^−1^ with a resolution of 1 cm^−1^. The samples were mixed with 90 wt% KBr and pressed into pellets for measurement.

### 2.3. Catalytic Performance Evaluation

The catalytic performances of the sulfonated carbon catalysts were evaluated by performing cellulose hydrolysis in the presence of these catalysts. Cellulose hydrolysis was tested using 100 mg of microcrystalline cellulose and 100 mg of the sulfonated CNTs in 10 mL of ultrapure water. The mixture was sealed in a 25 mL stainless-steel autoclave and heated at 150 °C for 24 h. After cooling, the mixture was separated using ordinary filter paper. The solution was analyzed using a Shimadzu HPLC (High-performance liquid chromatography, Kyoto, Japan) system, equipped with an RID-20A detector and a Shodex SUGAR SP0810 column with ultrapure water as the mobile phase at a flow rate of 0.6 mL min^−1^, column temperature 85 °C, and injection volume 10 µL. The HPLC-RID chromatogram for sugar content analysis of cellulose hydrolysis result is shown in Figure S1. Detector calibration was performed using standard solutions of glucose (0.1–10 g L^−1^), cellobiose (0.01–1 g L^−1^), and HMF (0.01–1 g L^−1^), along with other relevant sugar products, to obtain linear calibration curves. Cellulose conversion was calculated based on the carbon content of soluble products determined by Shimadzu TOC-L CSH TOC (Kyoto, Japan) analyzer, assuming complete conversion of cellulose carbon into soluble organics. Glucose yield and glucose selectivity were calculated using Equations (1) and (2), respectively.

(1) Glucose yield %=Conc.of glucose (gl)×V ml×6180 (gmol)

(2) $$Glucose\;selectivity\;\left(\%\right)=\frac{Glucose\;yield\;}{Cellulose\;convertion}\left(\%\right)$$

In Equation (1), the factor 6 represents the number of carbon atoms in each anhydro glucose unit (C_6_H_10_O_5_) of cellulose, which is used to normalize the glucose yield to glucose equivalents.

## 3. Results and Discussion

**Figure 2:**
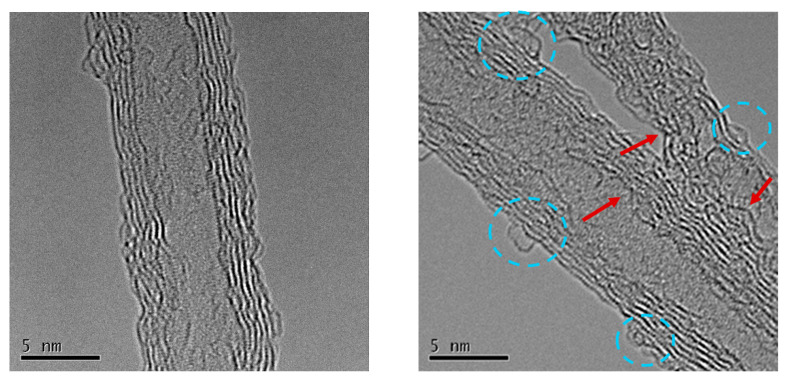
TEM image of original CNTs and treated CNTs (45 min treatment with 0.5 M Na_2_S_2_O_8_ solution). Blue circle: functionalized site; red arrowhead: kink.

As shown in Figure 2, TEM revealed that the tubular morphology of the CNTs was well preserved after the Na_2_S_2_O_8_ treatment. Kink refers to a type of structural instability, in which the fibers deform and form a curved or bent band. The treated CNTs had no notable damage sites [22]. The inner and outer diameters remained nearly unchanged at approximately 3.8 ± 0.4 nm and 7.5 ± 0.8 nm, respectively, confirming that the mild aqueous oxidation did not disrupt the graphitic backbone. However, surface roughening, kinks, and amorphous carbon layers were observed on the treated samples, indicating localized oxidative damage likely induced by radical attack. These defects suggest partial etching or rehybridization of outer wall carbon atoms [11]. Meanwhile, nitrogen adsorption measurements (Figure S2 and Table S1) showed a slight decrease in specific surface area and mesopore volume (10–100 nm), consistent with partial pore blockage by newly introduced functional groups.

**Figure 3:**
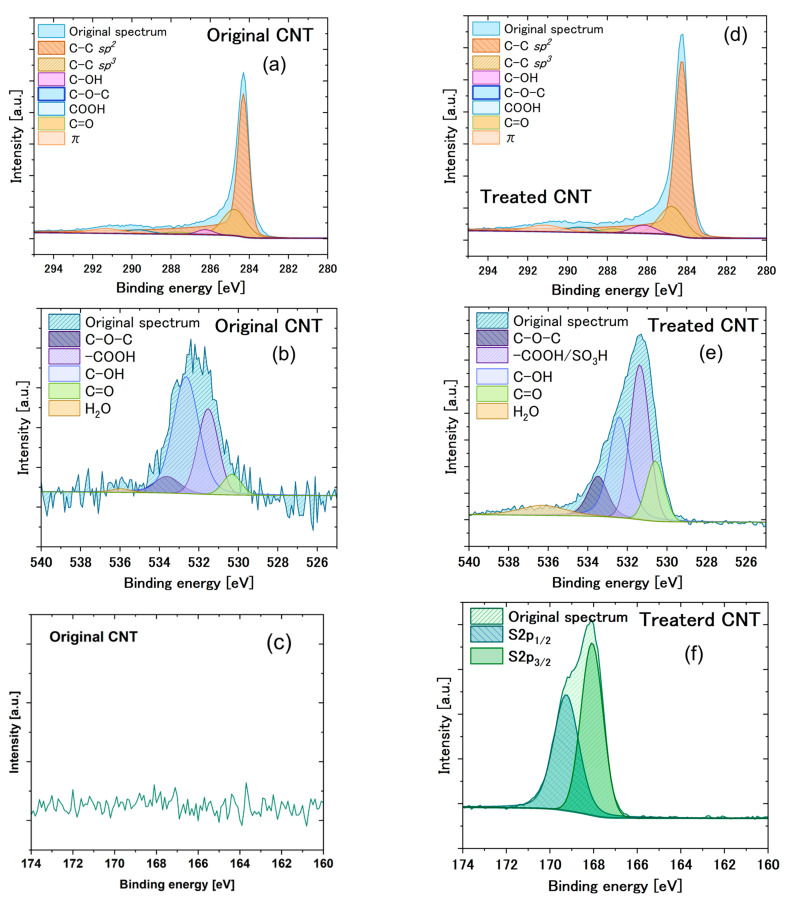
C 1s, O 1s, and S 2p XPS spectra of (**a**–**c**) initial CNT; C 1s, O 1s, and S 2p XPS spectra of (**d**–**f**) CNT after 45 min treatment by 0.5 M Na_2_S_2_O_8_ solution.

Elemental mapping (Figure S3) confirmed homogeneous distributions of carbon, oxygen, and sulfur across the treated CNTs. High-resolution XPS spectra (Figure 3) provided further insight into the surface functionalization. The emergence of a S 2p peak at 168 ± 0.2 eV is characteristic of sulfonic group (–SO_3_H) or sulfate group (–OSO_3_H) (Figure 3f) [23].

As shown in Figure 3a, the main peak in the C 1s is attributed to the C–C (*sp2*) and C–C (*sp3*) groups, which are located at 284.6 ± 0.2 eV and 284.8 ± 0.2 eV, respectively [24], and correspond to graphitic carbon. The hydroxyl group (C–OH) is located at 286.4 ± 0.2 eV, while the carbonyl group (C=O) and carboxylic group (O=C–OH) are located at 289.6 ± 0.2 eV and 287.9 ± 0.2 eV, respectively [25]. As shown in Table S1, the increased contributions from oxygen in the C 1s spectrum are primarily due to the enhancement of the C=O, –COOH, and –OH groups. This suggests that the production of these functional groups is the primary factor contributing to the observed increase in oxygen content.

In the O 1s spectrum (Figure 3b,e), the intensity of the O 1s peak for the treated carbon was significantly higher than that for the original untreated carbon, indicating the generation of numerous oxygen-containing functional groups during treatment process. The main enhancement in the O 1s spectrum the peak at 531.6 ± 0.2 eV, which is attributed to the presence of –COOH and –SO_3_H groups [26]. This shows that the expected production of –OH and –COOH groups, as well as the formation of –SO_3_H groups, contributed to the increased oxygen content on the carbon surface. Based on binding energies, characteristic O1s peaks are observed at 531.0 ± 0.2 eV, 532.9 ± 0.2 eV, and 534.0 ± 0.2 eV, corresponding to C=O, C–OH, and C–O–C, respectively [27,28,29].

The presence of sulfonic groups was further confirmed by FTIR analysis (Figure S4). All spectra exhibited characteristic vibrations of surface functional groups, including C=O (carbonyl or carboxyl) stretching at 1701 cm^−1^, C=C stretching of aromatic domains at 1602 cm^−1^ [30], and CH_2_ stretching at 2928 cm^−1^ [31]. In the spectra of treated CNTs, additional bands appeared from 1032 cm^−1^ to 1171 cm^−1^ [31,32], which are assigned to the O=S=O and S–O stretching modes of –SO_3_H groups, respectively.

**Figure 4:**
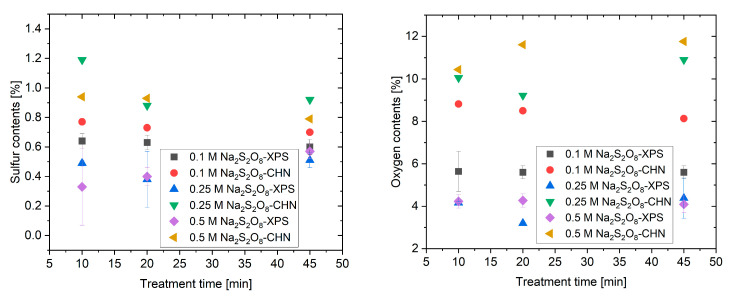
Elemental density variation of treated carbon; (**left**) sulfur content variation on the carbon material; (**right**) oxygen content variation on the carbon material.

The surface elemental densities of sulfur and oxygen were quantified by integrating the corresponding peak areas in the XPS survey spectra (Figure S5), while CHNS elemental analysis provided complementary information on the bulk composition. Quantitative analysis of the XPS and CHNS results (Figure 4) revealed that the treated CNTs reached a maximum sulfur content corresponding to an acid group density of 0.34 mmol g^−1^. This value exceeds that of sulfonated CMK-3 (0.23 mmol g^−1^) and other carbon materials prepared by conventional hydrothermal methods [33,34]. The –SO_3_H/–OSO_3_H group density on the carbon surface increased with treatment time at higher Na_2_S_2_O_8_ concentrations, whereas the change was less significant at lower concentration. Prolonged treatment time caused a gradual decline in bulk sulfur content, likely due to the partial removal of grafted groups. These findings confirm that moderate oxidant concentration and reaction time are crucial for maximizing surface functionalization without excessive oxidation. In addition, increasing treatment time resulted in slightly higher oxygen content, while sulfur incorporation reached a plateau beyond 10 min. Boehm titration confirmed a significant rise in total acid density compared with pristine CNTs, verifying that surface sulfonation occurred rapidly under these mild conditions (Figure S6). The bulk elemental ratios were consistently higher than the surface values, implying partial diffusion of oxidizing species into the inner layers of the CNT network.

**Figure 5:**
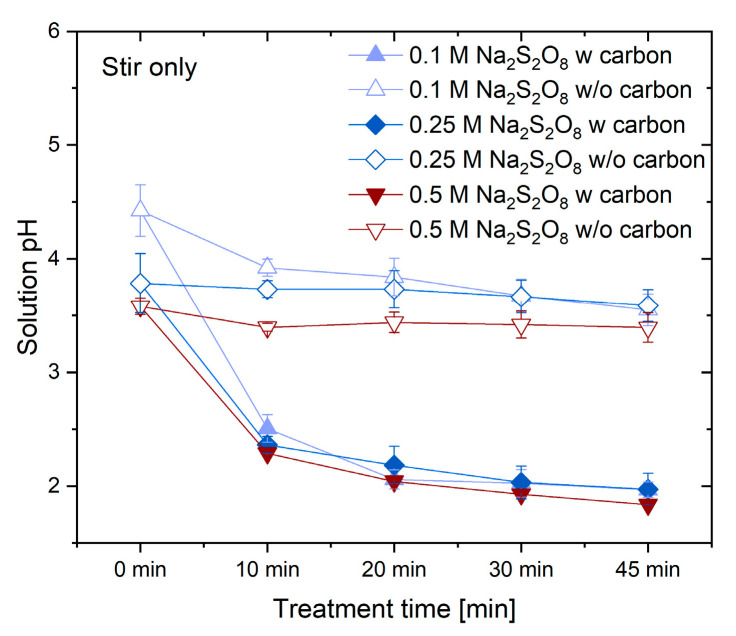
Solution concentrations variation with/without carbon treatment times.

To elucidate the mechanism of the persulfate activation reaction during catalyst synthesis, pH variations were recorded under different conditions (Figure 5). In the absence of carbon materials, the Na_2_S_2_O_8_ solution exhibited minimal pH change. In contrast, the addition of CNTs induced a pronounced pH drop from 4.4–3.6 to about 1.9 within 45 min, indicating carbon-assisted activation of persulfate. The following reactions account for this behavior.

This decrease in pH can be explained by the activation of the Na_2_S_2_O_8_ solution. Activated Na_2_S_2_O_8_ is commonly used in water purification processes because it can generate ∙SO_4_^−^. In this case, carbon appears to facilitate the activation of Na_2_S_2_O_8_, promoting the generation of ∙SO_4_^−^ radicals and reducing the solution pH, as illustrated by the following reactions [18]:

S_2_O_8_^2−^ + M–COOH → 2 ∙SO_4_^−^ + M–COO∙ + HSO_4_^−^

S_2_O_8_^2−^ + M–COOH → 2 ∙SO_4_^−^ + M–CO∙ + HSO_4_^−^

The generated ∙SO_4_^−^ radical can interact with the carbon surface to form –OSO_3_H groups, which contributes to the enhanced production of sulfur-containing functional groups on the carbon surface. Additionally, ∙SO_4_^−^ radicals can produce ∙OH radicals via secondary reactions. A byproduct of this process is the generation of H^+^ ions, reducing the pH of the solution [35,36]:

∙SO_4_^−^ + H_2_O → ∙OH + SO_4_^2−^ + H^+^

∙OH radicals generated in this process oxidize carbon material [37]:

∙OH + M → M–OH

∙OH + M–OH → M–COOH

In addition to ∙OH production, removal of the –OSO_3_H group on the carbon surface contributes to pH reduction. As shown below, HSO_4_^−^ ion production decreases solution pH [38]:

M–OSO_3_H → M=O + HSO_4_^−^

These radicals not only functionalize the carbon surface but also contribute to the oxidative restructuring that leads to the observed amorphous outer layer.

**Figure 6:**
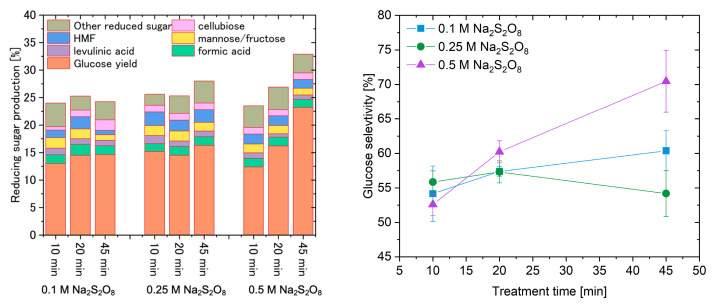
(**left**) Reducing sugar yield in the cellulose hydrolysis process with different solution concentrations and carbon treatment times; (**right**) glucose yield selectivity in the cellulose hydrolysis process. Carbon treatment times: 10 min, 20 min, 45 min. The hydrolysis conditions were 150 °C for 24 h. The glucose yield and cellulose conversion using the original carbon were 1.3% and 6.3%, respectively.

The catalytic performance of treated CNTs is shown in Figure 6 (left). The results confirm that the treated CNTs effectively catalyze cellulose hydrolysis. The glucose yield increased progressively with longer treatment times. Hydrolysis using catalysts treated with Na_2_S_2_O_8_ solutions of three different concentrations produced similar total reducing-sugar yields. The highest glucose yield and cellulose conversion were 23.18% and 31.60%, respectively. Compared with CNTs sulfonated by a conventional hydrothermal treatment which yielded 30.8% cellulose conversion and 24.8% glucose yield (Table S4), the Na_2_S_2_O_8_-treated catalyst achieved comparable performance under much milder, acid-free conditions. As shown in Figure 6 (right), the catalysts prepared with longer treatment times exhibited higher glucose selectivity. The observed decrease in glucose selectivity at higher Na_2_S_2_O_8_ concentration and short treatment time is likely due to the rapid formation of highly oxidative –SO_3_H groups. These groups may facilitate not only cellulose hydrolysis but also promote secondary decomposition of glucose into organic acids and other degradation products. In contrast, at longer treatment durations, the proportion of –SO_3_H groups decreases while other oxygen-containing groups such as –COOH and –OH increase. These milder acidic functionalities may suppress over-reactions and contribute to improved glucose selectivity, as observed in samples treated for 45 min. This trend suggests that both the type and density of surface functional groups significantly influence the balance between hydrolysis and glucose degradation pathways.

Catalyst durability was further examined through recyclability tests (Table S2). The glucose yield decreased by approximately 20.7% after the second run, accompanied by leaching of ~110 ppm SO_4_^2−^ ions—corresponding to 29.4% sulfur loss. This degradation is attributed to the limited thermal stability of –SO_3_H and –OSO_3_H groups at 150 °C [15]. CNTs prepared by hydrothermal sulfonation exhibited a similar trend (Table S4), with an initial glucose yield of 24.8% and a decline to 12.5% upon reuse. Although the acid-treated CNTs initially provided higher activity due to greater surface proton density (0.36 mmol g^−1^ SO_3_H; Table S3), the persulfate-treated CNTs demonstrated comparable catalytic stability while being synthesized under significantly milder, acid-free conditions. Moreover, when compared with previously reported sulfonated carbon catalysts and commercial solid acids (Table S5), the present system achieves competitive glucose yields under hydrothermal conditions, without the use of concentrated acids, elevated temperatures, or plasma activation. These comparisons highlight the operational and environmental advantages of the persulfate-mediated sulfonation route presented in this study. Although sulfur leaching remains a limitation, it provides a clear direction for future research aimed at enhancing the thermal stability of grafted functional groups. Overall, the persulfate-treated CNTs offer a promising platform for developing sustainable solid acid catalysts.

## 4. Conclusions

In summary, we developed a rapid and entirely acid-free approach for producing sulfonated carbon catalysts through ambient-temperature activation of sodium persulfate. The carbon surface mediates the in situ generation of sulfate and hydroxyl radicals, enabling efficient grafting of –SO_3_H and –OSO_3_H groups without any thermal or acidic treatment. The resulting CNT-based catalyst achieved up to 0.34 mmol g^−1^ of acid-site density and exhibited notable performance in cellulose hydrolysis, with a maximum conversion of 31.6% and glucose yield of 23.2%. Compared with traditional acid-based sulfonation routes, this route achieves comparable catalytic performance under ambient and acid-free conditions, with greatly reduced energy consumption and environmental burden. Although partial sulfur leaching was observed during reuse, this mild persulfate-driven functionalization provides a green and scalable alternative for the preparation of carbon-based solid acids, offering new opportunities for sustainable biomass conversion and catalytic applications.

## Figures and Tables

**Table 1:** Treatment conditions for CNTs sulfonation.

Na_2_S_2_O_8_ Concentration (mol/L).	Treatment Time (min)	Solution Temperature (°C)	CNTs Mass (mg)	Solution Volume (mL)
0.1/0.25/0.5	10/20/45	20	300	50

## Data Availability

The original contributions presented in this study are included in the article/Supplementary Material. Further inquiries can be directed to the corresponding author.

## Supplementary Materials

The following supporting information can be downloaded at: https://www.mdpi.com/article/10.3390/ma18215031/s1. References [39,40,41,42] are cited in the Supplementary Materials.
