# Supplementary material for: Green Technique for Producing Carbon-Based Catalysts for Cellulose Hydrolysis
Source: Materials (Basel). 2025 Nov 5;18(21):5031. doi: 10.3390/ma18215031 (PMC12608686; doi:10.3390/ma18215031)
Supplement: Supplementary file 1 [file materials-18-05031-s001.zip › materials-3953822-supplementary.pdf]

Green technique for producing carbon-based catalysts for cellulose hydrolysis

Siqi Deng[*a], Kaixun Yao[b], Manabu Kodama[c], Oi Lun Li[d], Nozomi Takeuchi[b]

[a] *Department of Electrical Engineering, Tohoku University*

[b] *Department of Electrical and Electronic Engineering, Institute of Science Tokyo*

[c] *Department of Mechanical Engineering, Institute of Science Tokyo*

[d] *School of Materials Science and Engineering, Pusan National University*

[*]*Corresponding author: deng@tohoku.ac.jp*

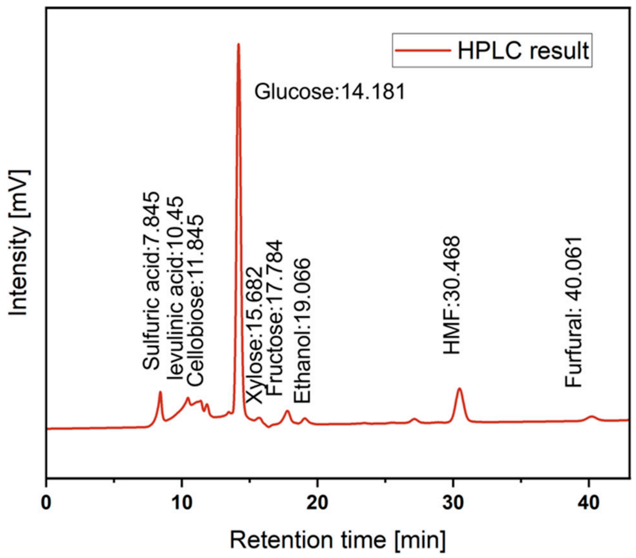

Figure S1: HPLC-RID chromatogram for sugar content analysis of cellulose hydrolysis result.

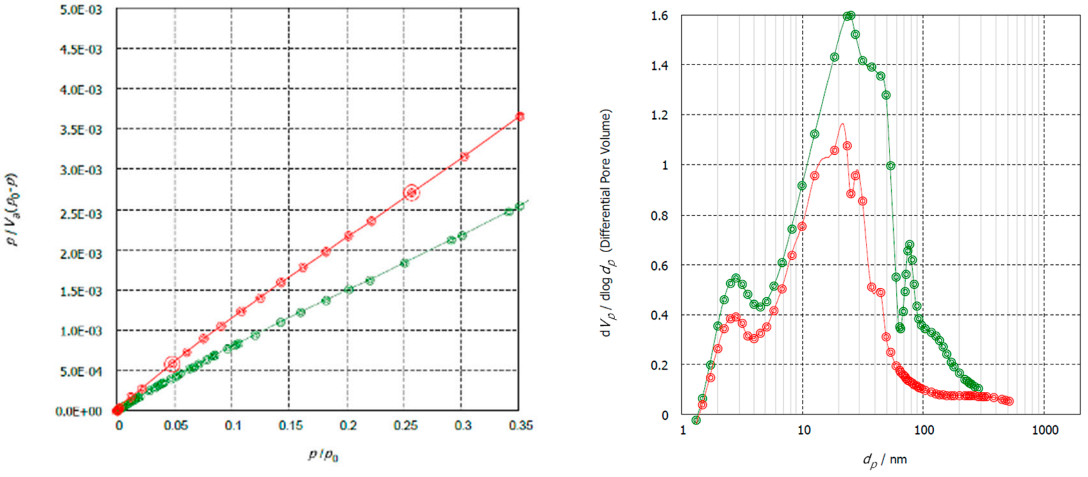

Figure S2: BET plot (left) and BJH plot (right) of original CNT (green line) and treated CNT (red line); Treatment condition: 45 min treatment with 0.5 M $Na_2S_2O_8$ solution.

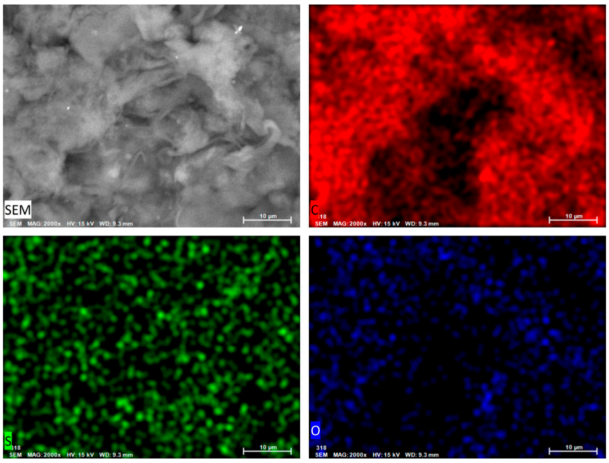

Figure S3: the SEM-EDS image of treated carbon (45 min treatment with 0.5 M $Na_2S_2O_8$ solution). Red: carbon; green: sulfur; blue: oxygen.

Table S1: Surface area, XPS peak assignment and atomic percentage (at. %) for original CNT and treated CNT (45 min treatment with 0.5 M $Na_2S_2O_8$ solution)

Sample	Surface area (m^2 g^{-1})	Total C 1s at.%	Total O 1s at.%	Total S2p at.%	Contributions from O to C 1s spectra at. %			
					C-OH	C-O-C	COOH	C=O
Original CNT	519	98.50	1.50	0.00	2.33	0.01	2.72	1.51

Treated CNT	424	91.11	7.63	1.26	3.54	0.06	2.79	2.11

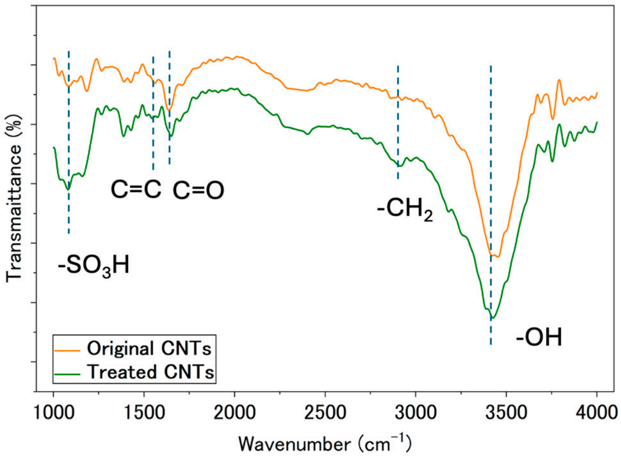

Figure S4: FTIR spectra of original and treated carbon materials (45 min treatment with 0.5 M $Na_2S_2O_8$ solution)

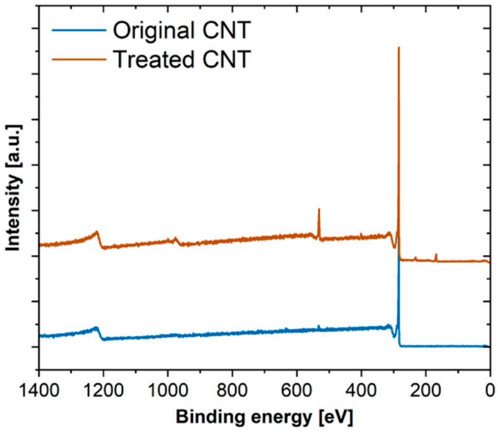

Figure S5: Survey XPS spectra of original and treated carbon materials (45 min treatment with 0.5 M $Na_2S_2O_8$ solution)

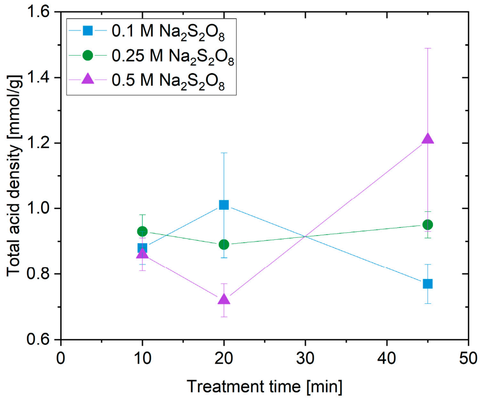

Figure S6: the variation of the total acid density of treated carbon with treatment time. (The total acid density of original CNT is 0.22 mmol g^{-1})

Table S2: The glucose yield and cellulose conversion after the 2nd run hydrolysis; the SO_4^{2-} in the solution after each hydrolysis. (CNT was treated with 0.25 M $Na_2S_2O_8$ solution for 10 min)

	Glucose yield [%]	Cellulose conversion [%]	SO_4^{2-} ion in the solution after hydrolysis [ppm]
1st run	15.9	25.5	113.5
2nd run	12.6	18.5	110.5

Table S3: Sulfonation results of CNTs treated by hydrothermal method.

Catalyst	Sulfonation Temperature (°C)/ Time (h)	SO_3H / COOH &OH density (mmol g-1)	Total acid densities (mmol g-1)
CNTs	150/10	0.36/1.10	1.46

Table S4: The glucose yield and cellulose conversion after the 2nd run hydrolysis; the SO_4^{2-} in the solution after each hydrolysis. (CNTs treated by hydrothermal method)

	Glucose yield [%]	Cellulose conversion [%]	SO_4^{2-} ion in the solution after hydrolysis [ppm]
1st run	24.8	30.8	141.9
2nd run	12.5	16.9	109.2

We evaluated thermal durability by isothermal oven heating followed by gravimetry. 30 mg of sample was heated in air at 150, 200, and 250 °C for 1 h at each step, cooled to room temperature, and weighed on a balance. The mass loss (%) at each temperature was calculated relative to the mass before that step. Pristine CNTs were measured as a control under identical conditions.

The treated CNTs exhibited 6.7 %, 3.3 %, and 13.0 % mass loss after 1 h at 150, 200, and 250 °C, respectively, compared with 6.7 %, 6.7 %, and 10.0 % for pristine CNTs. The larger loss of the treated sample at 250 °C (extra ~3 % absolute) indicates partial decomposition of grafted –SO_3H/–OSO_3H groups, while the similar behavior at 150 °C is consistent with removal of physisorbed water/volatiles in both materials. The small difference at 200 °C likely reflects experimental scatter and competing adsorption/desorption processes. Overall, these observations support the expected onset of sulfonic-group degradation around 200–250 °C, aligning with literature reports for sulfonated carbons[1,2].

Table S5: Comparison of the catalytic performance of treated carbon catalyst.

Catalyst & method	Preparation conditions	Total acid-site density (mmol g^{-1})	Cellulose hydrolysis condition (temperature, time; substrate; mass ratio of cellulose:catalyst)	Cellulose conversion (%)	Glucose yield (%)
This work	0.5 M $Na_2S_2O_8$, 45 min	1.52	150 °C, 24 h, H_2O, 1:1	31.60%	23.18%
Plasma-assisted sulfonated CNTs [3,4]	Plasma discharge with 1 M H_2SO_4 solution	3.47	150 °C, 15 h, H_2O, 9:10	37.4%	27.2%
Amberlyst-15 [5]	Commercial sulfonated	4.40	100 °C, 5 h, H_2O, 1:1	12%	-

	polymer resin				
Hydrothermal sulfonated active carbon[6]11/5/2025 11:28:00 AM	Concentrated H_2SO_4 (18 M), 150 °C, 10 h	0.72	150 °C, 24 h, H_2O, 1:1	-	21%

Reference:

1. Corrêa, A.P. da L.; Cardoso Bastos, R.R.; Filho, G.N. da R.; Roberto Zamian, J.; da Conceição, L.R.V. Preparation of Sulfonated Carbon-Based Catalysts from Murumuru Kernel Shell and Their Performance in the Esterification Reaction. **2020**, doi:10.1039/D0RA03217D.
2. Knauth, P.; Hou, H.; Bloch, E.; Sgreccia, E.; Di Vona, M.L. Thermogravimetric Analysis of SPEEK Membranes: Thermal Stability, Degree of Sulfonation and Cross-Linking Reaction. *Journal of Analytical and Applied Pyrolysis* **2011**, *92*, 361–365, doi:10.1016/j.jaap.2011.07.012.
3. Qin, L.; Ishizaki, T.; Takeuchi, N.; Takahashi, K.; Kim, K.H.; Li, O.L. Green Sulfonation of Carbon Catalysts via Gas–Liquid Interfacial Plasma for Cellulose Hydrolysis. *ACS Sustainable Chem. Eng.* **2020**, *8*, 5837–5846, doi:10.1021/acssuschemeng.9b07156.
4. Li, O.L.; Ikura, R.; Ishizaki, T. Hydrolysis of Cellulose to Glucose over Carbon Catalysts Sulfonated via a Plasma Process in Dilute Acids. *Green Chem.* **2017**, *19*, 4774–4777, doi:10.1039/C7GC02143G.
5. Rinaldi, R.; Palkovits, R.; Schüth, F. Depolymerization of Cellulose Using Solid Catalysts in Ionic Liquids. *Angewandte Chemie International Edition* **2008**, *47*, 8047–8050, doi:10.1002/anie.200802879.
6. Lai, D.; Deng, L.; Li, J.; Liao, B.; Guo, Q.; Fu, Y. Hydrolysis of Cellulose into Glucose by Magnetic Solid Acid. *ChemSusChem* **2011**, *4*, 55–58, doi:10.1002/cssc.201000300.
